# Consensus on quality indicators to assess the organisation of palliative cancer and dementia care applicable across national healthcare systems and selected by international experts

**DOI:** 10.1186/1472-6963-14-396

**Published:** 2014-09-17

**Authors:** Jasper van Riet Paap, Myrra Vernooij-Dassen, Rose-Marie Dröes, Lukas Radbruch, Kris Vissers, Yvonne Engels, on behalf of the IMPACT research team

**Affiliations:** Scientific Institute for Quality of Healthcare (IQ healthcare), Radboud university medical center, P.O. Box 9101, 6500, HB Nijmegen, The Netherlands; Kalorama Foundation, Nijmegen, The Netherlands; Department of General practice & Elderly care medicine and Department of Psychiatry, VU University Medical Centre, P.O. Box 7057, 1081, BT Amsterdam, The Netherlands; Department of Palliative Medicine, Universitätsklinikum Bonn, and Department of Palliative Care, Malteser Hospital Bonn, Sigmund-Freud-Street 25, 53127 Bonn, Rhein-Sieg, Germany; Department of Anaesthesiology, Pain and Palliative Medicine, Radboud university medical center, P.O. Box 9101, 6500, HB Nijmegen, The Netherlands

**Keywords:** Palliative care, Quality indicators, Dementia, Cancer, Europe

## Abstract

**Background:**

Large numbers of vulnerable patients are in need of palliative cancer and dementia care. However, a wide gap exists between the knowledge of best practices in palliative care and their use in everyday clinical practice. As part of a European policy improvement program, quality indicators (QIs) have been developed to monitor and improve the organisation of palliative care for patients with cancer and those with dementia in various settings in different European countries.

**Method:**

A multidisciplinary, international panel of professionals participated in a modified RAND Delphi procedure to compose a set of palliative care QIs based on existing sets of QIs on the organisation of palliative care. Panellists participated in three written rounds, one feedback round and one meeting. The panel’s median votes were used to identify the final set of QIs.

**Results:**

The Delphi procedure resulted in 23 useful QIs. These QIs represent key elements of the organisation of good clinical practice, such as the availability of palliative care teams, the availability of special facilities to provide palliative care for patients and their relatives, and the presence of educational interventions for professionals. The final set also includes QIs that are related to the process of palliative care, such as documentation of pain and other symptoms, communication with patients in need of palliative care and their relatives, and end-of-life decisions.

**Conclusion:**

International experts selected a set of 23 QIs for the organisation of palliative care. Although we particularly focused on the organisation of cancer and dementia palliative care, most QIs are generic and are applicable for other types of diseases as well.

## Background

Europe faces a huge challenge with a population that is rapidly aging in the coming decades. It is estimated that the incidence and prevalence of cancer will increase by about 20% and the prevalence of dementia will double before 2050 [1–4]. Although it concerns two totally different diseases with different care needs and disease trajectories, they do have a lot in common: they are often unnecessarily hospitalized, [5] have a high need for a multidisciplinary approach [6] and many suffer from symptoms which are partly the same, like pain [7, 8]. Higher survival rates of people with life-threatening and progressive chronic diseases will result in a larger number of patients that have multiple and complex health-threatening problems. Therefore, a growing number of patients will be in need of palliative care. However, a wide gap exists between the knowledge of best practice in palliative cancer and dementia care and its application in every day clinical practice [9].

As a first step in bridging this gap, it is important to assess current performance of palliative care in relation to its desired performance. Such an assessment of health care can be achieved by using quality indicators (QIs). QIs are evidence based, explicitly defined and measurable items that evaluate and describe structures, processes and outcomes of health care [10]. As such, they reflect the core elements of good clinical care. In day-to-day terms QIs can, for example, show whether pain is regularly being assessed using a validated tool; or whether a general practitioner is timely informed about a patient’s situation before or directly after discharge from hospital [11, 12]. QIs can help trace potential problems or confirm good quality of care and can be used to guide quality improvement processes [10]. They have been used effectively to assess and improve hospital care, [13] primary care, [14] and dementia care [15, 16]. Several studies have also developed QIs to improve the structures and process needed for the delivery of good quality palliative cancer or dementia care [11, 12, 17, 18]. However, these studies were performed five or more years ago, developed large sets of QIs, ranging from 56 to 142 QIs. Furthermore, none of these sets were widely implemented in everyday clinical practice.

The objective of this study was to integrate existing sets of QIs into one generic set that can be used to assess and improve the organisation of palliative care in different services and countries. The study was conducted within the framework of the European IMPACT project (**IM**plementation of quality indicators for **PA**lliative **C**are s**T**udy) [19].

## Methods

A modified RAND Delphi procedure was used to develop a set of QIs, [20] which is considered an accepted methodology to develop QIs [10]. Typically, a RAND Delphi procedure consists of a written and a face-to-face round [20]. In this study, four written rounds and one face-to-face round were performed to reach consensus about the essential aspects regarding the organisation of palliative care.

### Panellists

The IMPACT consortium consists of experts of the pan-European research group on detection and timely INTERvention in DEMentia (Interdem) and the European Association for Palliative Care (EAPC), all of whom are stakeholders in their country in palliative cancer and/or dementia care. The research team was invited to use their networks to purposefully select panellists for the modified RAND Delphi procedure. A key selection criteria was that the expert had to have extensive knowledge about palliative care, cancer care or dementia care. Additionally, experts had to be able to communicate in English (both verbally and non-verbally) and were planning to attend the 2012 Congress of the European Association of Palliative Care (EAPC) in Trondheim, Norway. Project partners nominated national and international experts in palliative cancer and dementia care. All nominated experts were approached via email (n = 50), forty experts from twelve countries agreed to participate in the modified RAND Delphi procedure (Table 1). All participants provided written informed consent. About half of them were experts in palliative cancer care and the other half in dementia care. Twenty-two panellists were active clinicians in this field (e.g. physician, nurse, psychologist, etc. currently involved in direct-patient care), the others were researchers.

**Table 1:** **Panellists per country**

Country	Researcher	Clinician
AU	1	
BE	1	2
CA		1
CH		2
DE	2	3
ES	1	1
IT		1
NL	8	3
NO	3	
PO		1
UK	2	7
US		1
Total =	18	22

AU: Australia, BE: Belgium, CA: Canada, CH: Switzerland, DE: Germany, ES: Spain, IT: Italy, NL: The Netherlands, NO: Norway, PO: Poland, UK: United Kingdom, US: United States.

### Selecting a preliminary set of QIs

A search for existing sets of QIs was conducted in PubMed. The search strategy was limited to English literature and consisted of various search terms that referred to subject-specific keywords describing palliative care (combined using “or”), as well as (“and”) the assessment of care using QIs (combined using “or”). Synonyms and medical subheading terms were used to fully include relevant literature (see Table 2).

**Table 2:** **Overview of search terms**

Palliative care	Quality indicators
Terminal care	Quality assurance
Hospice care	Quality measurement
Cancer care	Quality assessment
Dementia care	

Subsequently, references of key papers describing sets of QIs were hand searched. Additionally, consortium members of the IMPACT project were asked to nominate national and international sets of QIs on palliative cancer and dementia care they considered important. Two researchers (YE & JvRP) subsequently reviewed all of the identified QIs independently to determine if the QIs assessed the structure and process of palliative care and to structure them according to the domains of the recommended framework for the organisation of palliative care of the Council of Europe [21].

### First written Delphi-round

For the first written Delphi-round (April 2012), panellists received a personal invitation for an online questionnaire. To reduce the large number of identified QIs, panellists were asked to nominate one QI per domain of palliative care [22]. Those QIs that were nominated by the panellists were included in the second round of the modified RAND Delphi procedure.

### Second written Delphi-round

In the second round (May 2012), also via an online questionnaire, panellists were asked to rate QIs on a 9-point Likert scale for clarity (1 = not clear at all; 9 = very clear), usefulness (1 = not useful at all; 9 = very useful), to rephrase unclear and to add missing QIs. They were instructed to rate a QI high on usefulness if it: 1) corresponded with a basic quality level; 2) referred to a higher quality level that would be met only in very good practices; or 3) was associated with an innovative quality level which is exceptional at the moment, but could become the optimal quality level in the near future [23]. They were asked to give a low rating on usefulness if a proposed QI: 1) was too ambiguous or represented an unrealistically high quality level; 2) did not correspond with the material, social or cultural conditions of the situation in their country; or 3) was not in accordance with the regulations of palliative cancer and dementia care in their country [23].

### Third interactive Delphi-round

A consensus meeting was organised during the EAPC Congress June 7, 2012 in Trondheim, Norway. The meeting was chaired by an independent researcher with the aim to reach consensus on the QIs on which there was disagreement or where the median score was between 4 and 6 in the second Delphi round. Participants received a rating sheet on which the median ratings of the second Delphi-round of all experts were visible. Participants were given 30 minutes to rate the adapted QIs for usefulness. Next, per QI, participants were asked to raise their hand if they had rated usefulness 6 or less. If at least nine (30%) participants raised their hand, [20] the QI was discussed until consensus was reached.

### Fourth written Delphi-round

After the consensus-round, the remaining indicators were fed back to the panellist with the purpose to validate the changes that were made (September 2012).

### Fifth written Delphi round

In the final step of the QI development process, QIs were operationalised by the research team into questions that could be used by healthcare professionals to assess their organisation of palliative care and identify areas for improvement. During this process, it appeared that some QIs were inappropriate or not measurable (e.g. too time consuming to answer them appropriately). All QIs were therefore rated for necessity by the IMPACT research team (October 2012), representing both clinicians and researchers that also took part in the modified RAND Delphi procedure. QIs that were considered not necessary after this round, were omitted from the list.

### Analysis

QIs with a median rating on the usefulness scale of 7, 8 or 9 without disagreement were considered to have face validity. Disagreement was defined as: 30% or more of the panellists rated a single QI in the 1–3 tertile and more than 30% in the 7–9 tertile. QIs scored with a median of 1–3 without disagreement were not considered to have face validity. Because panellists had rated QIs high on usefulness, only QIs with median ratings of 8 or 9 were considered face valid for the second Delphi-round. Only QIs that were rated valid by all panellists were included in the final set [20].

### Ethical considerations

The Medical Ethics Committee of the district Arnhem-Nijmegen has declared that this study doesn’t fall within the remit of the Medical Research Involving Human Subjects Act (WMO) (registration number 2012/075). This means that this study can be carried out without an approval by an accredited medical ethics committee.

## Results

**Figure 1:**
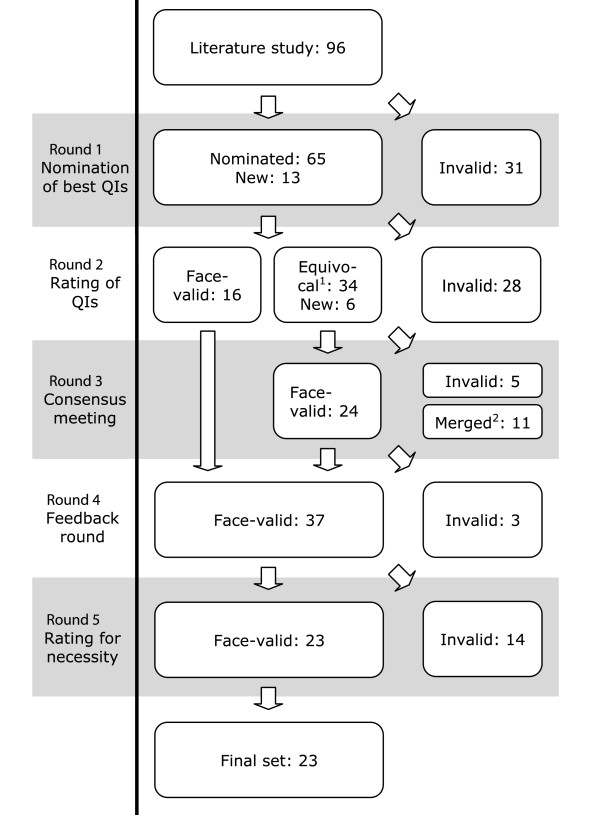
**Modified RAND Delphi procedure.** ^1^Equivocal is defined as all QIs on which there was no agreement: e.g. QIs with 30% or more of ratings in both the 1–3 tertile and the 7–9 tertile and all indicators with a median rating in the 4–6 tertile. ^2^At the end of round three, panelists agreed that 11 QIs could be merged.

650 QIs were selected from literature [11, 16, 18, 22, 24–54]. After having assessed these QIs, 554 were excluded because they were not about the organisation of palliative care or because of overlap; the remaining 96 QIs were included in a preliminary set of QIs (Figure 1). Of the 40 experts invited as panellists, 25 (63%) participated. In the first Delphi round, 65 of the 96 QIs were selected and 13 missing QIs were suggested in an open question in which panellists were asked if they missed any relevant QI. This resulted in an adapted list of 78 QIs, which were included in the second Delphi round.

In the second Delphi-round, 27 (67,5%) of the 40 invited panellists participated. Sixteen QIs were considered to have face validity, 28 were invalid, and six QIs were added to the list based on suggestions made by the panellists. The 40 QIs on which there was no agreement on, were included into the third Delphi round.

In the third Delphi-round, a consensus meeting, 29 (72,5%) of the 40 panellists participated. After having rated 40 QIs, one was excluded and 10 were discussed. Of the QIs that were discussed, six were accepted and four were excluded. Panellists also agreed to merge 11 QIs. Round three therefore resulted in 24 accepted QIs. The total list of QIs (QIs considered to have face validity in round two and three) comprised 40 QIs.

In the fourth Delphi-round, panellist provided feedback to the remaining set of QIs. This resulted in minor linguistic changes and the exclusion of three QIs because they were considered inappropriate by the majority of panellists. The resulting set of QIs, therefore, consisted of 37 QIs. This list was critically assessed by members of the IMPACT consortium for their necessity (round five). Fourteen QIs were considered to be overlapping, inappropriate, or not measurable in palliative care. The final list of QIs, therefore, consisted of 23 QIs, covering seven domains (Table 3). Key findings can be summarized as follows:

**Table 3:** **Overview of QIs**

***1. Access to palliative care***	
***1a. Access and availability***	
1.	A specialist palliative care team* is available 24/7.
2.	Specialist palliative care* advice is available 24/7 to professionals delivering palliative care.
3.	Bereaved relatives and/or professionals involved in care of a person in need of palliative care are offered support during the bereavement process if they need or wish to have support.
***1b. Out of hours care***	
4.	Opioids are accessible and available for persons in need of palliative care 24/7.
5.	Co-analgesics* for symptom control are available to treat persons in need of palliative care 24/7.
***1c. Continuity of care***	
6.	An (electronic) file of a person in need of palliative care is accessible to professionals in charge of the person 24/7.
7.	At each transition between care settings, comprehensive information (including care goals and care plan) of a person in need of palliative care is be transferred to the professional(s) in charge in the next setting.
8.	The professional in charge of the person is informed before a person in need of palliative care is discharged home or sent to the next setting.
9.	Persons in need of palliative care have an assigned contact person who maintains regular contact with the person and their families, and ensures coordinated delivery of health and social care.
***2. Infrastructure***	
10.	Specialised equipment (e.g. anti decubitus mattresses, suction equipment, stoma care, oxygen delivery, drug administration pumps, hospital beds, etc.) is available to persons in need of palliative care.
11.	Single bedrooms are available for persons who are dying and who wish to have one.
12.	Family members and friends are able to visit the dying person without restrictions of visiting hours.
13.	There are facilities for relatives to stay overnight with their dying relative.
14.	There is a private area for saying goodbye to the deceased, nearby or on the ward/unit where the person died.
***3. Assessment tools***	
15.	There is a regular assessment of pain and other symptoms using a validated instrument*.
***4. Personnel***	
***4a. Team***	
16.	The multidisciplinary team* that delivers palliative care services consists of at least:
	a) a physician and nurse;
	b) and has access to one or more of the following professionals: physiotherapist, psychologist, occupational therapist, social worker, chaplain, dietician.
17.	There is a weekly multidisciplinary meeting with at least the physician and nurse in charge of the person in need of palliative care to review treatment and care plans.
***4b. Sharing information***	
18.	The file of the person in need of palliative care contains documentation of a discussion with the person or representative (if the person lacks capacity e.g. is unable to communicate) about:
	a) medical condition;
	b) goals for treatment;
	c) the physical*, psychosocial and spiritual needs of the person and family caregiver;
	d) an advance directive or advanced care plan;
	e) end-of-life decisions*;
	f) the intention to return home or to another facility from the place where the person is currently staying.
***5. Documentation of clinical data***	
***5a. Clinical records***	
19.	The file of the person in need of palliative care contains a medication list that is accessible to the professionals caring for the person.
***5b. Timely documentation***	
20.	Within 48 hours of admission to the service, the file of the person in need of palliative care contains documentation of the initial assessment of:
	a) pain and other symptoms, using a validated instrument*;
	b) psychosocial and spiritual needs;
	c) persons preferences, wishes and needs;
	d) capacity to be involved in the decision making process.
***6. Quality***	
21.	Family and caregiver experiences of the palliative care service are assessed/evaluated/recorded.
22.	An end-of-life care pathway (such as the Liverpool Care Pathway) was used for the last 3 days of life of a person in need of palliative care.
***7. Education***	
23.	All professionals that deliver palliative care services receive accredited training in palliative care, appropriate to their discipline.
NB	Where person is stated, one can also read patient.
**Glossary**	
**Palliative care team**: A home palliative care team provides specialised palliative care* to patients who need it at home (or home replacing institute) and support to their families and carers at the patient’s home. They also provide specialist advice to general practitioners, family doctors and nurses caring for the patient at home. The core team of a home palliative care team consists of four to five full-time professionals and comprises physicians and nurses with specialist training, a social worker and administrative staff. The home palliative care team works in close collaboration with other professionals so that the full range of multi-professional team work can be realised in the home-care setting. (Source: Radbruch L, Payne S: **White paper on standards and norms for hospice and palliative care in Europe: part 2.** *European Journal for Palliative Care* 2010, **17:**22**–**33).	
A hospital palliative care support team provide specialist palliative care* advice and support to other clinical staff, patients and their families and carers in the hospital environment. They offer formal and informal education, and liaise with other services in and out of the hospital. A hospital palliative care support team is composed of a multiprofessional team with at least one physician and one nurse with specialist palliative care training. The team should have ready access to other professionals working in liaison with it, including bereavement specialists, chaplains, dietitians, therapists, oncologists, pharmacists, physiotherapists, psychiatrists, psychologists, social workers and speech and language therapists. (Source: Radbruch L, Payne S: **White paper on standards and norms for hospice and palliative care in Europe: part 2.** *European Journal for Palliative Care* 2010, **17:**22**–**33).	
A **team** is hereby defined as a group of people organized to work together, which consists of at least a nurse and a physician.	
**Specialist palliative care**: Specialist palliative care is provided by specialised services for patients with complex problems not adequately covered by other treatment options. Specialist palliative services require a team approach, combining a multiprofessional team with an interdisciplinary mode of work. Team members must be highly qualified and should have their main focus of work in palliative care. (Source: Radbruch L, Payne S: **White paper on standards and norms for hospice and palliative care in Europe: part 2.** *European Journal for Palliative Care* 2010, **17:**22**–**33).	
**Co-analgesics**: An adjuvant (or co-analgesic) is a drug that in its pharmacological characteristic is not necessarily primarily identified as an analgesic in nature, but that has been found in clinical practice to have either an independent analgesic effect or the additive analgesic properties when used with opioids. (Source: Khan M.I.A., Walsh D., Brito-Dellan N: **Opioid and Adjuvant Analgesics: Compared and Contrasted.** *AM J HOSP PALLIAT CARE* 2011, 28(5) 378–383)	
**Validated instrument**: Instruments such as the Visual Analogue Scale (VAS) or the Numeric Rating Scale (NRS) that can be used to indicate the severity of the patient’s pain or other symptom. (Source: Ahmedzai S, Gómez-Batiste X, Engels Y, Hasselaar J, Jaspers B, Leppert W, Menten J, Mollard JM, Vissers K: *Assessing Organisations to Improve Palliative Care in Europe.* Nijmegen: Vantilt Publishers; 2010).	
**End-of-life decisions**: End-of-life care may be used synonymously with palliative care or hospice care, with end of life understood as an extended period of one to two years during which the patient/family and health professionals become aware of the life-limiting nature of their illness. End-of-life care may also be understood more specifically as comprehensive care for dying patients in the last few hours or days of life. Either way, the patient preserves his/her self-determination regarding the power of decision on place of care, treatment options and access to specialist (palliative) care. End-of-life decisions are all the decisions made by the patient/family and health professionals regarding this last phase of a patient’s life, e.g. decisions that may influence the time of death, either prolonging life (or prolonging dying) or shortening life (or let patients die). (Source: Radbruch L, Payne S: **White paper on standards and norms for hospice and palliative care in Europe: part 1.** *European Journal for Palliative Care* 2010, **16(6):**278**–**289).	
**Physical needs**: For example if the patients physical symptoms require certain needs, such as special bed, walking aid, etc.	

### Access to palliative care

The availability of a dedicated palliative care team was considered important by almost all panellists in the Delphi procedure. They explicitly stated that palliative care services should not only be available during office hours, but at all times (day, evening, night and weekends). Furthermore, specific elements of palliative care were considered important, such as the availability of opioids and anticipatory medications for symptom control, as well as the availability of bereavement support.

Almost all panellists also rated important the accessibility of the medical record to health care professionals, timely transfer of information between settings (including when transferring or discharging patients). Panellists also considered an assigned contact person, who maintains regular contact with patients and their families, useful.

### Infrastructure

The infrastructure of the place where palliative care is provided, such as a single bed hospital room, was rated important. Access to equipment (such as anti-decubitus mattresses, suction equipment, etc.), required to provide palliative care, was considered important. Panellists also rated high consensus for facilities for relatives to visit, stay overnight, and a private area for saying goodbye to the deceased. There was no agreement on QIs that aimed to control waiting time or waiting list, i.e. these aspects were not considered to be unique for palliative care and therefore not important.

### Assessment tools

Regular assessment of pain and other symptoms was rated as a valid quality criterion, though it was commented that a validated instrument might not always be available, particularly for specific patient groups (e.g. for persons with advanced dementia).

### Personnel

There was agreement on the need for a multidisciplinary team, which should consist of at least a physician and nurse, and have access to a range of supporting disciplines, such as: physiotherapist, psychologist, occupational therapist, social worker, dietician, and chaplain. Panellists also rated a regular multidisciplinary team meeting important.

### Documentation of clinical data

Panellists recognized the importance of having a well-structured medical record. However, a QI about the structure of the medical record was not considered important by the panellists. Panellists only considered the inclusion of a medication regimen in the medical record important. They also considered a timely assessment (within 48 hours) of pain and other symptoms, psychosocial and spiritual needs, patient preferences, wishes and needs, and the patient's capacity to be involved in the decision making process as important.

Furthermore, almost all panellists rated the documentation of communication on the medical condition, goals of treatment, physical, psychosocial, and spiritual needs of the patient and their relatives, intention to return home, advanced directive, and end-of-life decisions as important.

### Quality and safety

Panellists rated the QI about assessing the experiences of care givers with the palliative care service important. Secondly, a QI about the quality of care, assessing the use of an end-of-life care pathway within the last three days of life, was also considered useful.

### Education

QIs about the staff’s learning objectives and a program for specialised and/or continuing medical education about the physical, psychosocial, and spiritual needs of a patient in need for palliative care were not rated important. There was also no agreement on disease-specific education for staff members, but panellists considered palliative care training specified to the professiona’ls background important.

## Discussion

With the help of a modified five-round RAND Delphi-procedure, we were able to develop an internationally validated set of QIs for the organisation of palliative care with high face validity as judged by experts in the field of cancer and dementia care. The final set provides 23 quality aspects regarding the accessibility of the service, its infrastructure, the use of symptom assessment tools, management of personnel, documentation of clinical data, quality of care, and education. Of these 23 QIs, one was identical to the original one (*Family members and friends are able to visit the dying person without restrictions of visiting hours*), [12] two were new (*Family and caregiver experiences of the palliative care service are assessed/evaluated/recorded* and *An end-of-life care pathway (such as the Liverpool Care Pathway) was used for the last 3 days of life of a person in need of palliative care*) and 20 were rephrased QIs. Panellist agreed not to formulate disease-specific QIs for the organisation of palliative care, since our set of QIs provide information about the organisation of services and not about the care provision itself. This might explain why so many QIs were rated face-valid for as well the organisation of cancer as dementia palliative care: regarding aspects as access to and 24 h availability of specialist palliative care or transferring information between settings, the specific condition of the patient (advanced cancer, dementia, or even COPD or heart failure) is not relevant, making our set of QIs much more broadly applicable. Therefore, these QIs are generically applicable and can be used in different settings. Thereby, they can also be used for (cross-)national comparisons and to identify best practices regarding the organisation of palliative care in other services and countries.

In the recent literature several sets of QIs for palliative care have been identified [11, 12, 17, 38, 55]. For example, Pastrana et al. used a nominal group technique to identify indicators for the assessment and evaluation of palliative care [38]. However, they primarily focused on the German health care system, which makes this set difficult to apply in an international context [38]. Pasman et al. conducted a literature review, and identified 142 QIs in 16 studies [11]. However, this set also has not been developed within an international context and it does not focus on the organisation of palliative care [11]. An update of this review, published in 2013, included a further 187 QIs, bringing the total to 326 QIs, with still few QIs about the organisation of palliative care [55]. Around the same time, Woitha et al. developed a set of 56 QIs [12, 17]. Woitha et al. conducted two written Delphi rounds, leaving little room for discussion, while we conducted a consensus round with extensive opportunity to discuss the QIs. Secondly, they included professionals from different European countries only, while we also included professionals from countries such as Canada, Australia and the USA, making the set of QIs presented here globally applicable. Thirdly, they focused on the organisation of palliative care in general and did not specifically consider the organisation of palliative care for patients with dementia, like was done in the present study.

A recently published White paper defining optimal palliative care in older people with dementia, [56] described several recommendations on palliative care treatment for persons with dementia. All of their recommendations that can be translated to the organisation of care, like the use of assessment tools, multidisciplinary meetings, bereavement support and about specialist palliative care teams, are represented in our QI set.

Another quality indicator, suggested by the World Health Organization as part of the framework programme on non-communicable diseases has been the focus of attention recently. This QI is being proposed to describe access to palliative care by assessing morphine equivalent consumption per death from cancer [57, 58]. However, this QI has been criticized as it might have provided flawed information due to inaccuracies in the underlying data base and the unavailability of national cancer registries [59]. Instead of assessing palliative care on a global level, we aim to assess whether palliative care services meet a basic quality level or higher quality level that would be met only in very good practices. Our set of QIs can therefore be used as internal QIs by health care providers (professionals and managers) to monitor and improve their service. They can also be used to describe and rank services according to performance, but this should not lead to a quality rating, as there may be good reasons for the differences in performance with the QI (e.g. different organisational structure).

Using our QIs as an external quality assessment tool will therefore make them unfit for their task [60]. Berwick et al. summarized this as ‘measuring for improvement is not measuring for judgement’ [61]. An ongoing intervention in 40 palliative care services in Europe, including hospitals, hospices, nursing homes and primary care settings, performed as part of the IMPACT project, in which this set of QIs is used as starting point to assess the organisation of palliative care, will evaluate the feasibility and discriminatory power of the QIs in relation to improving the organisation of palliative care in the participating services.

### Strengths and limitations

Strengths of this study were that we used a large international group of panellists for our Delphi procedure, who were actively involved in palliative care (such as members of the European Association for Palliative Care). Secondly, by organising our consensus meeting during the EAPC Congress in Trondheim, Norway, key persons active in the fields of palliative care and dementia care were able to contribute extensively to the discussion of the Delphi procedure. Thirdly, the multidisciplinary character of palliative care was represented by the panellists (e.g. physicians, nurses, psychologists, etc.) involved in the Delphi procedure. Furthermore, half of them were professionals active in dementia care. Fourth, combining QIs for the organisation of services that provide care to palliative patients with cancer and those that provide such care to patients with dementia is unique. Our QIs can therefore be used in different settings.

A limitation of this study is that this set of QIs might not be comprehensive. Because an international, generic set of QIs was developed, some QIs that were important in only one or a few countries were excluded from the list. For that reason, important national or setting-specific QIs must be added when the set is used in a specific country. Secondly, this set of QIs is only related to the organisation of palliative care. Outcome and patient-related outcome measures were not included because they address a distinct purpose in measuring quality of palliative care. Thirdly, participants of the modified RAND Delphi procedure were selected because of their knowledge about palliative care, cancer care or dementia care. Because some experts of two large European networks (EAPC and InterDem) are part of the IMPACT consortium, they were also selected as participant for the modified RAND Delphi procedure (n = 18). Although not all countries were represented (like France) and others were overrepresented (like the Netherlands), the experts covered 12 countries from three continents, covering different health care systems and types of organisation of palliative care. Pilot testing the set of QIs in those countries and continents that were not represented in this study will reveal whether they are applicable in these countries too. Fourthly, unfortunately, there were no patient representatives involved as panellist. Testing the final set of QIs will therefore also have to incorporate their views on the basic quality level or higher quality level that would be met only in very good services.

## Conclusion

International experts selected a set of 23 QIs for the organisation of palliative care that can be implemented in daily practice in order to demonstrate that organisations are providing high quality and effective palliative care or to identify areas for improvement.
